# Characterization of the stimulators of protein-directed ribosomal frameshifting in Theiler's murine encephalomyelitis virus

**DOI:** 10.1093/nar/gkz503

**Published:** 2019-06-10

**Authors:** Sawsan Napthine, Susanne Bell, Chris H Hill, Ian Brierley, Andrew E Firth

**Affiliations:** Division of Virology, Department of Pathology, Addenbrooke's Hospital, University of Cambridge, Cambridge, UK

## Abstract

Many viruses utilize programmed –1 ribosomal frameshifting (–1 PRF) to express additional proteins or to produce frameshift and non-frameshift protein products at a fixed stoichiometric ratio. PRF is also utilized in the expression of a small number of cellular genes. Frameshifting is typically stimulated by signals contained within the mRNA: a ‘slippery’ sequence and a 3′-adjacent RNA structure. Recently, we showed that −1 PRF in encephalomyocarditis virus (EMCV) is *trans*-activated by the viral 2A protein, leading to a temporal change in PRF efficiency from 0% to 70% during virus infection. Here we analyzed PRF in the related Theiler's murine encephalomyelitis virus (TMEV). We show that 2A is also required for PRF in TMEV and can stimulate PRF to levels as high as 58% in rabbit reticulocyte cell-free translations and 81% during virus infection. We also show that TMEV 2A *trans*-activates PRF on the EMCV signal but not *vice versa*. We present an extensive mutational analysis of the frameshift stimulators (mRNA signals and 2A protein) analysing activity in *in vitro* translation, electrophoretic mobility shift and *in vitro* ribosome pausing assays. We also investigate the PRF mRNA signal with RNA structure probing. Our results substantially extend previous characterization of protein-stimulated PRF.

## INTRODUCTION

Programmed ribosomal frameshifting (PRF) is a gene expression mechanism whereby a proportion of translating ribosomes are stimulated to shift into an alternative reading frame at a specific site during the decoding of an mRNA (1). Ribosomes which frameshift produce an alternative protein product that is N-terminally coincident with the product of standard decoding but has a distinct C-terminal region encoded by either the +1 or the −1 reading frame depending on the type of frameshifting. In viruses, the most common type of PRF involves −1 nt tandem slippage of the P- and A-site tRNAs on the mRNA (−1 PRF). Many viruses use −1 PRF to express the viral polymerase at a set ratio with other components of the replication complex, with examples including HIV and other retroviruses, SARS and other coronaviruses, and many plant RNA viruses (1). Other viruses use −1 PRF to append an extension domain onto a proportion of their capsid proteins (e.g. (2,3)), or to express accessory proteins (e.g. (4,5)). A number of cellular genes also utilize −1 PRF in their expression, both in eukaryotes (6,7) and in bacteria (8,9).

In eukaryotes, sites of −1 PRF normally conform to an X_XXY_YYZ ‘slippery heptanucleotide’ shift site motif, where XXX represents any three identical nucleotides, YYY represents UUU or AAA, Z represents A, C or U, and underscores separate zero-frame codons. Such sites allow for P- and A-site anticodon:codon re-pairing following a −1 nt shift, except potentially at the wobble positions. It should be noted that the requirement for re-pairing is weaker in the P-site and a number of exceptions to XXX occur, such as GGU, GUU, GUC, GAA, GGA and UCC (1). While such slippery heptanucleotides may allow frameshifting of up to ∼1–2% (for some sequences), in order to achieve a high efficiency, an extra stimulator is required and this normally takes the form of a 3′ stable RNA stem–loop or pseudoknot structure separated from the shift site by a ‘spacer’ region of 5–9 nt. Structures of this type are thought to be located at the mRNA unwinding site of the ribosome entrance channel when their stimulatory effect is exerted (10). How the stimulatory RNAs function to promote −1 PRF is still uncertain, but accumulating evidence from prokaryotic counterparts indicates that the RNA structure impedes back rotation of the ribosomal small subunit, trapping the ribosome in a rotated or hyper-rotated state (11,12). This stalled state can be resolved either via spontaneous unwinding of the structure or via a −1 PRF which, by repositioning the structure within the mRNA entrance channel, is thought to allow for more efficient unwinding by the ribosome (11).

Intra-mRNA structures normally lead to a fixed ratio of frameshifting, ideal for controlling stoichiometry of different components of the replication complex or different structural proteins. Recent work with porcine respiratory and reproductive syndrome virus (PRRSV) (family: *Arteriviridae*) uncovered a new case of PRF where the stimulator involves, not an intra-mRNA structure separated from the shift site by a 5–9 nt spacer, but instead a protein binding site (CCCANCUCC) separated from the shift site by a 10-nt spacer (13–15). The viral protein nsp1β together with host poly(C) binding proteins (PCBPs) bind at this site, and the RNA:protein complex positioned at the leading edge of the ribosome when the decoding centre is positioned on the shift site is sufficient to stimulate ∼20% efficient −2 PRF and ∼7% efficient −1 PRF in virus-infected cells. Even higher levels (up to ∼50% −2 PRF) have been observed in a recombinant vaccinia virus/T7 polymerase expression system. More recently, a completely independent case of protein-stimulated PRF was identified in encephalomyocarditis virus (EMCV) (family *Picornaviridae*; genus *Cardiovirus*) where the viral 2A protein binds to an RNA stem–loop structure separated from the shift site by a 13-nt spacer (16–18). Ribosome profiling analyses showed that this RNA:protein complex strongly impedes ribosome progress but that this impediment can be relieved by ribosomes shifting to the −1 reading frame on the G_GUU_UUU shift site. Cellular levels of 2A build up strongly between 4 and 6 h post infection (p.i.), and this correlates with a switch in frameshifting efficiency from negligible levels at 2 and 4 h p.i. to ∼70% at 6 and 8 h p.i., making this one of the most efficient cases of −1 PRF in a mammalian cell system and the only case known to be temporally modulated.

To date, despite there being hundreds of identified cases of RNA-structure stimulated PRF, these are the only two known cases of protein-stimulated PRF. Our previous work on protein-stimulated PRF in genus *Cardiovirus* focused on EMCV. Another well-studied member of this genus is Theiler's murine encephalomyelitis virus (TMEV). The EMCV G_GUU_UUU frameshift site is conserved in TMEV, as is the presence of a 3′ stem–loop structure separated from the shift site by a 13-nt (EMCV) or 14-nt (TMEV) spacer. In TMEV, however, the stem–loop is more compact with a 10-nt loop compared to a 21-nt loop (albeit probably containing internal structure) in EMCV. Further, the TMEV 2A protein shares only ∼27% aa identity with the EMCV 2A protein. The late-timepoint PRF efficiencies in virus-infected cells have previously been measured at ∼70% in EMCV (by ribosome profiling) and 74–82% in TMEV (by metabolic labelling, which may be less accurate). However, the role of TMEV 2A in the stimulation of PRF on the TMEV mRNA has not been studied, nor has the ability of TMEV 2A to cross-activate EMCV PRF, or *vice versa*.

Here we investigate protein-stimulated PRF in TMEV. We show that the TMEV 2A protein stimulates frameshifting on the TMEV mRNA and to a level in rabbit reticulocyte lysate (RRL) cell-free translations considerably higher than observed previously with the EMCV 2A protein at the EMCV signal. Thus, TMEV may be a more tractable *in vitro* system for future structural and biophysical studies of 2A action. We show that TMEV 2A stimulates efficient PRF on both EMCV and TMEV mRNAs whereas EMCV 2A is active only on the cognate EMCV mRNA. Since 2A is known to interact with the viral L protein, we tested whether EMCV L has an effect on EMCV PRF but found none. We also performed an extensive mutational analysis of protein and RNA stimulators of TMEV PRF and tested the effects of these mutations in *in vitro* translation, electrophoretic mobility shift (EMSA) and *in vitro* ribosome pausing assays. The predicted TMEV RNA stem–loop structure was verified with chemical and enzymatic structure probing. Finally a 2A mutant defective in RNA binding was introduced into the virus genome and tested in the context of infection. Our results confirm the role of 2A in stimulating PRF at the TMEV signal and substantially extend previous characterization of cardiovirus frameshifting stimulators.

## MATERIALS AND METHODS

### Cells, recombinant viruses and plasmids

Cell lines were obtained from the European Collection of Authenticated Cell Cultures (ECACC) and tested for mycoplasma by PCR (e-Myco *plus* Mycoplasma PCR Detection Kit; iNtRON Biotechnology).

WT and mutant viruses are based on the GDVII strain of TMEV and were generated from the full-length infectious clone pSK-GDVII (a kind gift from the Robert Fujinami lab, University of Utah). The sequence of this clone is identical to GenBank accession number NC_001366.1 except for three nucleotide differences: G2241A (serine to isoleucine in VP2), A2390G (synonymous change in VP3), and G4437A (lysine to glutamine in 2B). Nucleotide coordinates herein are given with respect to NC_001366.1 (19). All constructs were prepared by standard PCR mutagenesis and recombinant DNA techniques and subcloned regions altered by mutagenesis were verified by DNA sequencing. All viruses were able to replicate in cell culture. The SS mutations do not alter the polyprotein amino acid sequence. EMCV sequences were obtained from pMC0 (developed by Ann Palmenberg, University of Wisconsin-Madison; (20)) which is identical to GenBank accession number DQ294633.1 in the region encoding 2A and the −1 PRF signal.

For *in vitro* frameshifting assays, we cloned a 105-nt sequence containing the G_GUU_UUU shift site flanked by 6 nt upstream and 92 nt downstream, or mutant derivatives, into the dual luciferase plasmid pDluc at the *Xho*I/*Bgl*II sites (21). The sequence was inserted between the *Renilla* and firefly luciferase genes so that firefly luciferase expression is dependent on −1 PRF. For the *in vitro* frameshifting assays with additional 3′ sequence added, NC_001366 nucleotides 4230–4436 (14 nt 5′ + G_GUU_UUU shift site + 186 nt 3′) followed by nucleotides 7500–8101 (last 480 nt of polyprotein ORF + entire 122-nt 3′ UTR) followed by 21 nt of poly(A) were cloned into vector pEGFP_C1 at the *Xho*I/*Bam*HI sites to generate pEGFP_C1-TGF. In this construct, the TMEV-encoded sequence is fused to the C-terminus of eGFP and the non-frameshift product also includes 160 amino acids corresponding to the C-terminus of the TMEV polyprotein. Construct pEGFP_C1-TG5 is the same except that the region comprising nucleotides 7500–8101 and the poly(A) tail was omitted.

For ribosomal pausing analysis, the EMCV or TMEV shift site flanked by 6 nt upstream and 86 nt downstream, or mutant derivatives, were cloned into pPS0 at the *Xho*I/*Pvu*II sites (22) to generate pPS-TMEV-WT, pPS-TMEV-SS and pPS-EMCV-SS. For the expression of recombinant 2A or L in *Escherichia coli*, the 2A or L coding sequences were amplified from pMC0 (EMCV) or pSK-GDVII (TMEV) and cloned into pGEX-6P-2 (GE Healthcare) at the *Bam*HI/*Xho*I sites. The expressed proteins, following removal of the GST moiety by PreScission Protease (a kind gift from Stephen Graham, University of Cambridge), have an additional five (GPLGS-) vector-derived residues at the N-terminus. Further, our early versions of EMCV 2A and TMEV 2A have a vector-derived C-terminal extension (-NSRVDSSGRIVTD in EMCV and -EFPGRLERPHRD in TMEV). All TMEV 2A mutants M1, M2, M3 (a.k.a. 2A-mut) and M4 have the C-terminal extension. All EMSAs use the C-terminally extended TMEV and EMCV 2As except where indicated otherwise in Figure 8C and D. Elsewhere (i.e. *in vitro* pausing and frameshifting assays) the WT EMCV and TMEV 2As have the authentic C-terminus, except where WT 2A is compared with mutants M1–M4 (Figure 7) where the C-terminally extended WT 2A was used for a rigorous comparison.

### *In vitro* transcription and generation of recombinant virus

RNA was transcribed using the Megascript T7 kit (Ambion) from *Bam*HI-linearized plasmids. Reactions were phenol/chloroform extracted, RNA desalted by centrifugation through a NucAway Spin Column (Ambion) and concentrated by ethanol precipitation. Purified RNA was used to transfect 35-mm dishes of BHK-21 cells using 1.2 μg RNA and 4 μl DMRIE-C reagent according to the manufacturer's instructions. Transfected cells were cultured in Dulbecco's modified Eagle's medium (DMEM) with high glucose and 1% fetal bovine serum (FBS) for 1 to 5 d depending upon how rapidly cytopathic effect (CPE) developed. Cultures were subjected to three rounds of freeze-thawing, cell debris removed by centrifugation for 5 min at 4000 *g* and the supernatant stored in aliquots at −80°C.

### Plaque assays

BHK-21 cells at 90% confluence in 6-well plates were infected with serial dilutions of virus stocks. Cells were washed with serum-free medium, overlaid with virus innoculum and incubated for 1 h at 37°C. Innocula were removed and replaced with 1.5% low melting point agarose (Invitrogen) containing DMEM containing 2% FBS. After 40 h incubation at 37°C, cells were fixed with formal saline and stained with 0.1% toluidine blue.

### Metabolic labelling and calculation of PRF efficiencies

BHK-21 cells were infected at a multiplicity of infection (MOI) of ∼5 in a volume of 150 μl in 24-well plates. After 1 h the inoculum was replaced with 1 ml DMEM containing 2% FBS. At 7 h p.i., cells were incubated for 1 h in methionine- and serum-free DMEM, and radiolabelled from 8 to 9 h p.i. with [^35^S] methionine at 100 μCi/ml (∼1100 Ci/mmol) in methionine-free medium. Cells were scraped into the medium, pelleted at 13 000 *g* for 1 min, washed twice by resuspension in 1 ml of ice-cold phosphate-buffered saline (PBS) and pelleted for 2 min at 13 000 *g*. Cell pellets were lysed in 35 μl 4× SDS-PAGE sample buffer and boiled for 5 min before analysis by 12% SDS-PAGE. Dried gels were exposed to X-ray films or to phosphorimager storage screens. Image analysis was carried out using ImageQuantTL 7.0, and the radioactivity in virus-specific products quantified.

The intensity for each WT virus product was measured, normalized by methionine content, and then by the mean value for VP3 and VP1 to control for lane loading. Next, to factor out differences in protein turnover besides unquantified processing intermediates, for each biological replicate the WT and 2A-mut values for VP3, VP1, 2B, 3C and 3D were normalized by corresponding values for SS mutant virus. Then the normalized values for 2B, 3C and 3D (i.e. products encoded downstream of the frameshift site) were averaged and divided by the average of the values for VP3 and VP1 (i.e. products encoded upstream of the frameshift site). This gives an estimate of the fraction of ribosomes that avoid a −1 PRF (Figure 9D). One minus this value estimates the PRF efficiency.

### Structure probing

Short, ^33^P-labelled RNAs (105 nt) containing the TMEV PRF region (shift site plus 29 nt upstream and 69 nt downstream) were prepared by T7 transcription of a PCR product generated using primers flanking the PRF region, with the 5′ primer containing the T7 RNA polymerase promoter sequence. Structure mapping, using 10 mCi/ml [γ-^33^P]ATP (PerkinElmer), was performed using a 5′-end-labelling procedure as described previously (23). All probing reactions were performed in a final volume of 50 μl containing ∼20 000 cpm 5′ [^33^P] end-labelled transcript, 2 mM MgCl_2_, 10 μg pig liver rRNA, and the relevant enzymatic or chemical probe. Products were analyzed on a 10% acrylamide/7M urea gel.

### Protein expression and purification

N-terminally glutathione-S-transferase (GST) tagged proteins were purified from *E. coli* BL21/DE3/pLysS cells. Overnight cultures inoculated from a single colony were used to inoculate expression cultures which were then grown at 37°C to an OD_600_ of 0.6. Protein expression was induced by addition of isopropyl β-d-1-thiogalactopyranoside (to 0.1 mM) and continued for overnight at 22°C after which cells were pelleted and resuspended in lysis buffer (1.4 mM β-mercaptoethanol, 0.5 mM MgCl_2_, 0.05% Tween 20, 20 mM Tris pH 7.5, 150 mM NaCl, DNase 1 U/ml and protease inhibitor 1 U/ml). Cell lysates were prepared by sonication (30 min on ice), and cleared by centrifugation (39 000 *g*, 4°C, 30 min). Proteins were purified using glutathione agarose resin (GE Healthcare) according to standard procedures (24), then dialysed against 50 mM Tris, pH 7.5, 100 mM KCl, 1 mM dithiothreitol (DTT), 0.05 mM ethylenediaminetetraacetic acid (EDTA) and 5% glycerol, quantified by Bradford assay (ThermoFisher Scientific), and stored at −80°C until required.

### Electrophoretic mobility shift assay

Short, ^32^P-labelled template RNAs containing the TMEV (59 nt) or EMCV (64 nt) PRF region (with slippery sequence precisely at the 5′ end) were prepared by T7 transcription of a PCR product generated using primers flanking the PRF region, with the 5′ primer containing the T7 polymerase promoter sequence. Radiolabelled RNAs were mixed with test proteins in 10 μl reactions in EMSA buffer (10 mM HEPES pH 7.6, 150 mM KCl, 2 mM MgCl_2_, 1 mM DTT, 0.5 mM adenosine triphosphate, 5% glycerol, 100 μg/ml porcine tRNA, 10 U RNase inhibitor ml^−1^). Test proteins were diluted in dilution buffer (DB) (5 mM Tris pH 7.5, 100 mM KCl, 1 mM DTT, 0.05 mM EDTA, 5% glycerol). For competition experiments, unlabelled competitor RNA was incubated with WT ^32^P-labelled RNA (10 nM) and 2A (0.7 μM). Reactions were incubated at 30°C for 10 min before promptly loading the mix onto non-denaturing, 4% acrylamide gels (acrylamide:bisacrylamide ratio 10:1). Gels were run at 175 V at room temperature until free and bound RNA species were resolved, then fixed for 15 min in 10% acetic acid, 10% methanol, dried and exposed to X-ray film and phosphorimager screen.

### *In vitro* translation

Frameshift plasmids based on pDluc were linearized with *Fsp*I and capped run-off transcripts generated using T7 RNA polymerase as described previously (25). TGF and TG5 RNAs were prepared by T7 transcription of PCR products generated using a 5′ primer containing the T7 polymerase promoter sequence upstream of the AUG of eGFP and appropriate 3′ primer sequences for TGF and TG5. Messenger RNAs were translated in nuclease-treated rabbit reticulocyte lysate (RRL) or wheat germ (WG) extracts (Promega) programmed with ∼50 μg/ml template mRNA. Typical reactions were of 10 μl volume and composed of 90% (v/v) RRL, 20 μM amino acids (lacking methionine) and 0.2 MBq [^35^S]-methionine. Reactions were incubated for 1 h at 30°C and stopped by the addition of an equal volume of 100 μg/ml RNase A in 10 mM EDTA, followed by incubation at room temperature for 20 min. Proteins were resolved by 12% SDS-PAGE and dried gels were exposed to X-ray film or to a Cyclone Plus Storage Phosphor Screen (PerkinElmer). The screen was scanned using a Typhoon FLA 7000 (GE Healthcare) in storage phosphor autoradiography mode and bands were quantified using ImageQuant™TL software (GE Healthcare). PRF efficiencies were calculated as [IFS1/MetFS1] / [IS/MetS + IFS1/MetFS1], where the number of methionines in the stop and −1 frameshift products are denoted by MetS and MetFS1 respectively, and the densitometry values for the same products are denoted by IS and IFS1, respectively. In the cases where both −1 and −2 frameshift products were measurable, PRF efficiencies were calculated as [IFS1/MetFS1]/[IS/MetS + IFS1/MetFS1 + IFS2/MetFS2] (for −1 PRF) and [IFS2/MetFS2] / [IS/MetS + IFS1/MetFS1 + IFS2/MetFS2] (for −2 PRF). Frameshifting assays were performed at least three times. A statistical analysis of a representative dataset (from Figure 7) is provided in the Supplementary Information.

### Ribosome pausing assays

WG *in vitro* translation reactions (30 μl) were supplemented with 1 μM of TMEV 2A, TMEV 2A-mut, EMCV 2A, or dialysis buffer, and programmed with mRNAs derived from *Ava*II-cut pPS-TMEV-WT, pPS-TMEV-SS or pPS-EMCV-SS. Reactions were incubated at 18°C for 5 min prior to the addition of edeine to 5 μM final concentration. Aliquots (1.5 μl) were subsequently withdrawn at set intervals, mixed with an equal volume of 100 μg/ml RNase A in 10 mM EDTA, and placed on ice. At the end of the time-course, products were resolved by 12% SDS-PAGE. The expected size of the ribosomal pause product was marked by translating a control mRNA produced from *Xho*I-cleaved pPS0.

## RESULTS

### TMEV 2A *trans*-activates PRF

**Figure 1.**
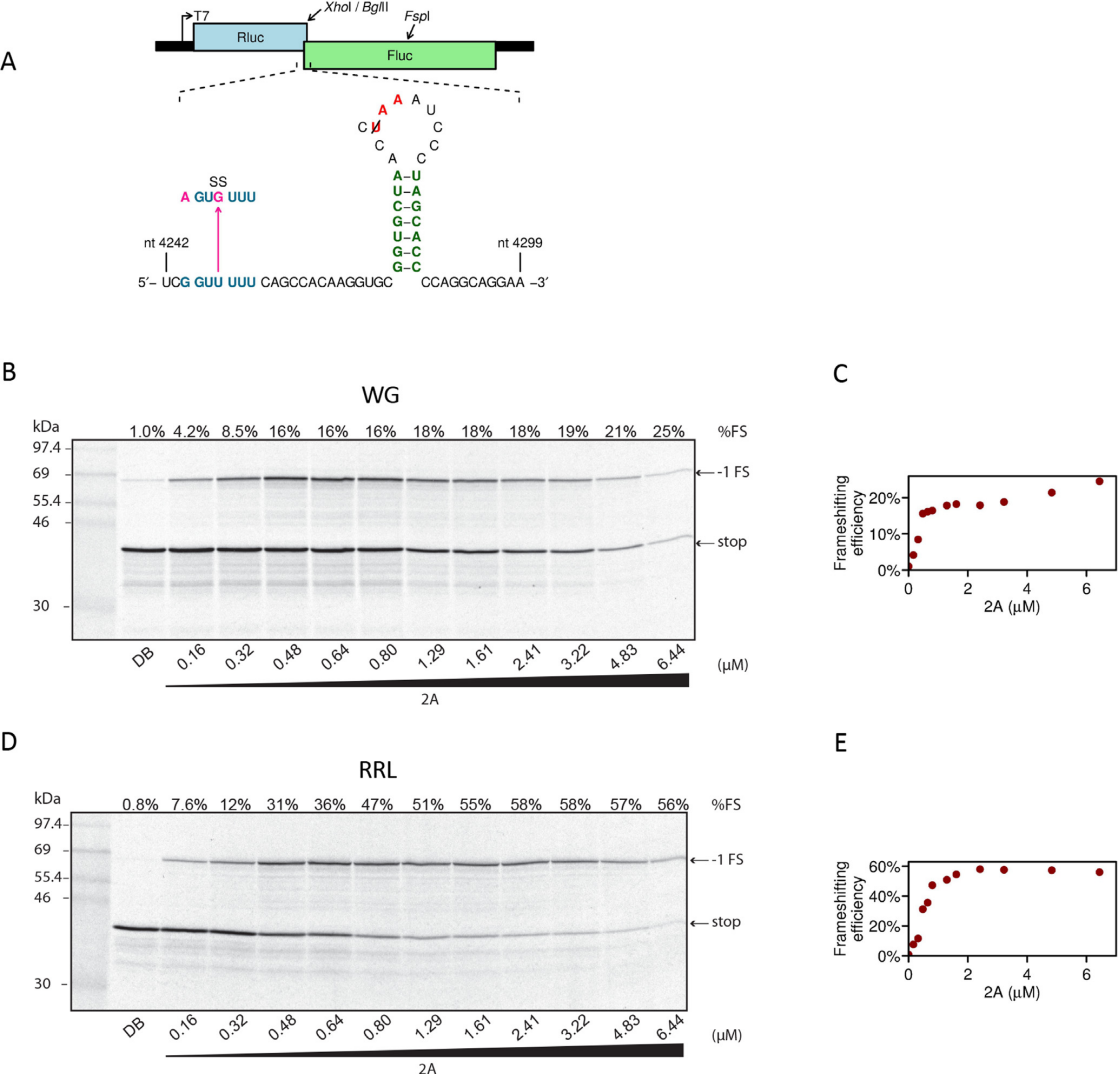
Effect of increasing concentrations of 2A on PRF efficiency. (**A**) Mutations introduced into the TMEV PRF signal in reporter plasmid pDluc. Nucleotide coordinates refer to the TMEV genome. All constructs (including WT) contain a U to C mutation in the loop to remove the −1 frame stop codon UAA (red). (B–E) RNAs containing the TMEV PRF signal were translated in WG (**B**) or RRL (**D**) in the presence of increasing concentrations of 2A, or 2A dialysis buffer (DB). Products generated by ribosomes that do not frameshift (stop) or that enter the −1 reading frame (−1 FS) are indicated. Frameshifting efficiencies estimated from densitometry are indicated above lanes. Markers are in lane 1. The PRF efficiencies are shown in (**C**) and (**E**).

**Figure 2.**
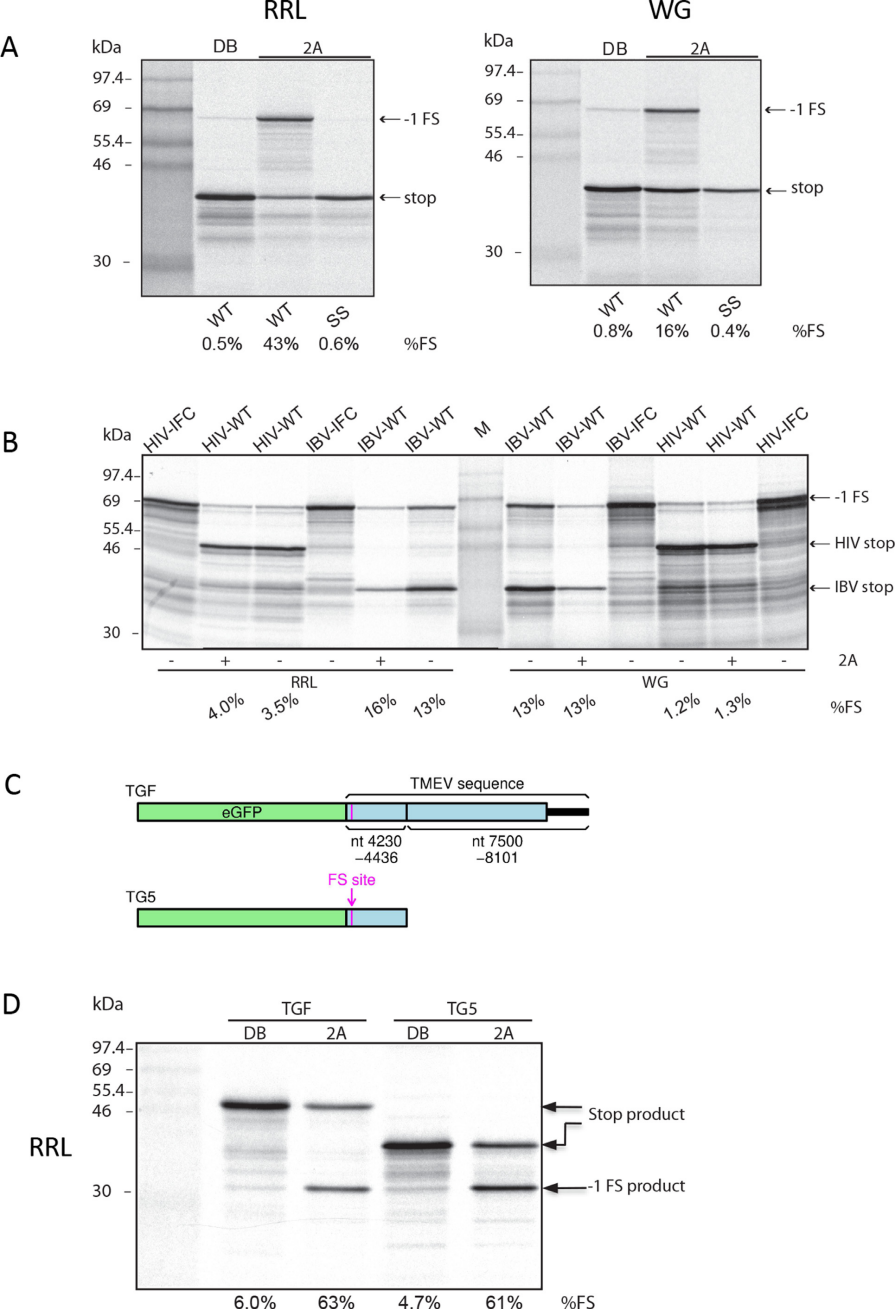
2A stimulation of PRF. (**A**) RNAs containing the TMEV PRF signal were translated in RRL (left) or WG (right) with 1.8 μM recombinant 2A (lanes 3–4) or with dialysis buffer (DB). SS indicates a shift site mutant. Products generated by ribosomes that do not frameshift (stop) or that enter the −1 reading frame (−1 FS) are indicated. Markers are in lane 1. (**B**) RNAs containing the IBV or HIV PRF signals were translated in WG or RRL with or without 1.8 μM recombinant TMEV 2A protein. IFC indicates in-frame controls showing the position at which the frameshift products migrate. Markers are in the middle lane. (**C**) Schematic of eGFP-based constructs TGF and TG5. TGF (‘TMEV GFP full’) contains the TMEV shift site (G_GUU_UUU) preceded by 14 nt 5′ and followed by 186 nt 3′, fused to the last 480 nt of the TMEV polyprotein ORF, the entire TMEV 3′ UTR and 21 nt of poly(A). TG5 (‘TMEV GFP 5′’) is similar but lacks the 480 nt + 3′ UTR + poly(A) region. (**D**) TGF and TG5 RNAs were translated in RRL with 1.8 μM recombinant 2A (lanes 3 and 5) or with dialysis buffer (DB; lanes 2 and 4). Markers are in lane 1.

Knowing already that EMCV 2A *trans*-activates PRF on the EMCV frameshift sequence, we first tested whether TMEV 2A *trans*-activates PRF on the TMEV frameshift sequence. We titrated recombinant TMEV 2A protein into a wheat germ extract (WG) cell-free translation system programmed with a dual luciferase-based reporter mRNA containing the TMEV PRF signal. Following previous work (16), we engineered a U to C change in the loop of the stimulatory RNA to remove the −1 frame UAA stop codon, so that ribosomes which frameshift read into the downstream reporter gene (Figure 1A). Increasing amounts of 2A led to a general inhibition of cap-dependent translation, as reported previously for EMCV 2A (Figure 1B) (26,27). When the reporter was translated in the absence of 2A, only ∼1% PRF was observed (Figure 1B, lane DB). However in the presence of 2A, efficient PRF was observed, to a level of ∼20% with increasing amounts of 2A (Figure 1B and C). Similar results were observed in RRL, except that the PRF efficiency plateaued at a higher level (∼58%; Figure 1D and E). As a control, we confirmed that inactivation of the slippery sequence (G_GUU_UUU to A_GUG_UUU mutation; SS mutant) reduced frameshifting to background levels in both WG and RRL (Figure 2A). To test whether 2A stimulation was specific to the cardiovirus PRF signal, reporter mRNAs containing the human immunodeficiency virus 1 (HIV; family *Retroviridae*) or infectious bronchitis virus (IBV; family *Coronaviridae*) frameshift signals (28) were translated in WG or RRL with or without recombinant TMEV 2A protein. For these signals, addition of 2A had no stimulatory effect on PRF efficiency (Figure 2B).

### PRF in TMEV is not modulated by more distal conserved 3′ sequences

Previously, in the context of a reporter construct, we tested the contribution to PRF of mRNA sequences immediately 3′ proximal to the slippery sequence (92 nt 3′ in (16) for TMEV and up to 125 nt 3′ in (17) for EMCV). However, it is possible that PRF is further modulated by more distal sequence elements, as occurs in luteoviruses where −1 PRF is stimulated by a 3′-proximal extended stem–loop structure which forms a kissing interaction with a second stem–loop positioned nearly 4 kb downstream (29).

To test for potential additional signals, we generated a new eGFP-based reporter mRNA (TGF; ‘TMEV GFP full’) containing the TMEV shift site (G_GUU_UUU) preceded by 14 nt and followed by 186 nt of TMEV sequence, fused to the last 480 nt of the TMEV polyprotein ORF, the entire TMEV 3′ UTR and 21 nt of poly(A) (Figure 2C). We also generated another reporter mRNA (TG5; ‘TMEV GFP 5′’) which was similar except that the 480 nt + 3′ UTR + poly(A) region was omitted. Our rationale for including these sequences was that (a) 3′ UTRs often contain regulatory elements, (b) our earlier analysis of synonymous site conservation in an alignment of TMEV-related cardiovirus sequences revealed conserved overlapping elements (potentially RNA signals) in the 3′ region of the polyprotein ORF (Figure 1B of (17)), and (c) although this analysis also suggested that there were no additional conserved elements 3′-proximal to the shift site other than what was already included in our original reporter construct above (containing the shift site and 92 nt of 3′-adjacent sequence) we reasoned that including additional TMEV-derived 3′-proximal sequence might reduce the potential for spurious base-pairing interactions between the 3′ stem–loop and either vector sequence in the original construct or the TMEV far 3′-sequences in the new construct, which might impact PRF efficiency.

Translation of these reporter mRNAs in RRL showed that both of the extended constructs permitted similar levels of frameshifting in the presence of 2A (63% for TGF and 61% for TG5) indicating that neither the conserved elements within the last 480 nt of the TMEV polyprotein ORF nor the TMEV 3′ UTR play a role in PRF stimulation (Figure 2D). These values were also similar to those observed above (∼58%) for mRNAs with only 92 nt instead of 186 nt of TMEV-derived sequence 3′-adjacent to the shift site.

### A CCC triplet in a 3′ stem–loop is essential for PRF

**Figure 3.**
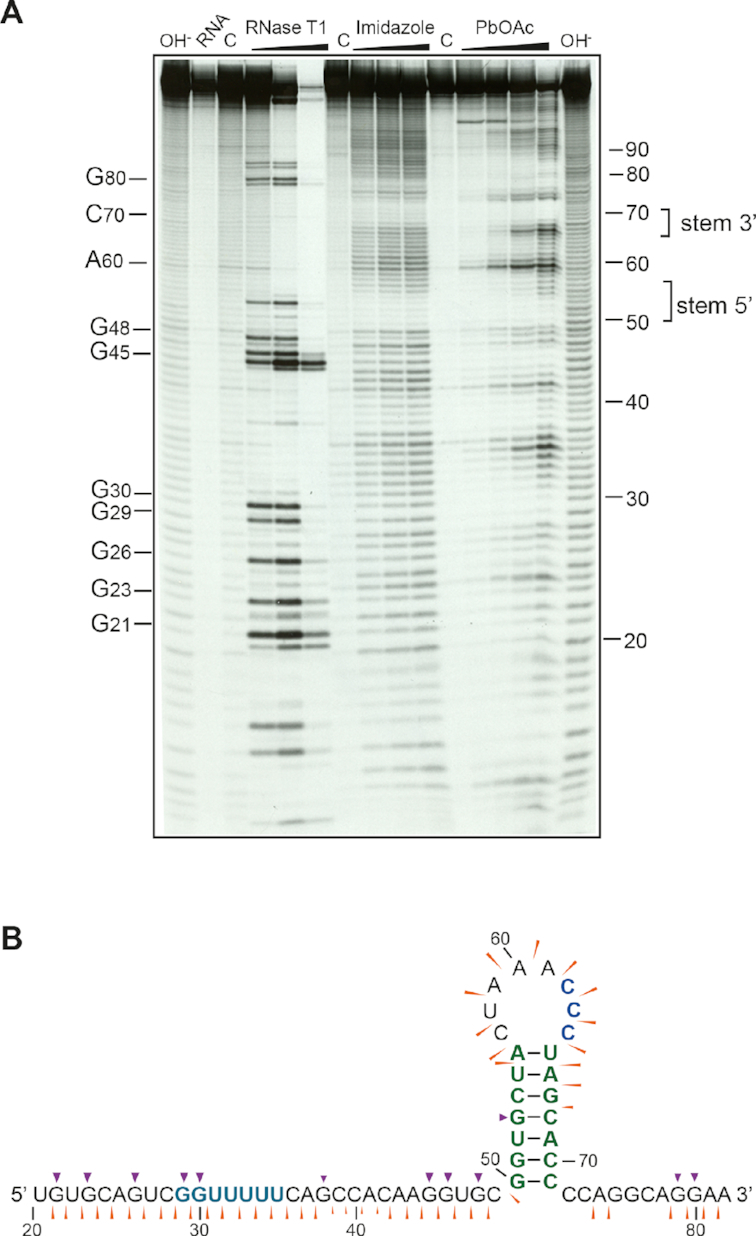
Structure probing of the 3′ stem–loop. (**A**) RNA was 5′-end-labelled with [γ-^33^P] ATP and subjected to limited RNase or chemical cleavage using structure-specific probes. Sites of cleavage were identified by comparison with a ladder of bands created by limited alkaline hydrolysis of the RNA (OH^−^; RNA heated to 100°C for 95 s [left lane] or 165 s [right lane]). Enzymatic probing employed RNase T1 (at 1, 10 and 100 U/mL) which preferentially cleaves after G bases in single-stranded regions. Chemical probing was with imidazole (2, 4 and 6 h exposure), or lead acetate (PbOAc; 10, 20 and 50 mM concentration in reaction), which show specificity for single-stranded regions. Uniquely cleaved nucleotides were identified by their absence in untreated control lanes (C). The regions corresponding to the 5′ and 3′ part of the stem–loop duplex are indicated. (**B**) The reactivities of RNase T1 (purple arrowheads) and imidazole (thin orange arrowheads) are indicated on a schematic of the TMEV PRF signal showing the evolutionarily conserved stem–loop structure (18). The size of the symbols is approximately proportional to the intensity of cleavage at that site.

In EMCV, TMEV and related cardioviruses, there is a conserved predicted 3′ RNA stem–loop structure separated from the shift site by a 13–14 nt spacer (Supplementary Figure S3A of (18)). A completely conserved CCC triplet in the loop is important for 2A binding in EMCV (18). The EMCV stem–loop structure is supported by enzymatic and chemical structure probing, and a mutational analysis has shown it to be critical for PRF stimulation (18,30). To provide support for the predicted stem–loop structure in TMEV we performed chemical and enzymatic structure probing (Figure 3). Short, ^33^P-labelled RNAs (105 nt) containing the TMEV PRF region (shift site plus 29 nt upstream and 69 nt downstream) were treated with chemical (imidazole, lead acetate; which show specificity for single-stranded regions) or enzymatic (RNase T1; cuts single-standed RNA after G residues) probes. The cleavage pattern of the RNA was generally consistent with the proposed stem–loop structure, although the upper portion of the 3′ arm of the proposed duplex stem was accessible to the single-stranded chemical probes. Single-stranded probe cleavage was especially evident in the slippery sequence and the A-triplet in the loop region.

**Figure 4.**
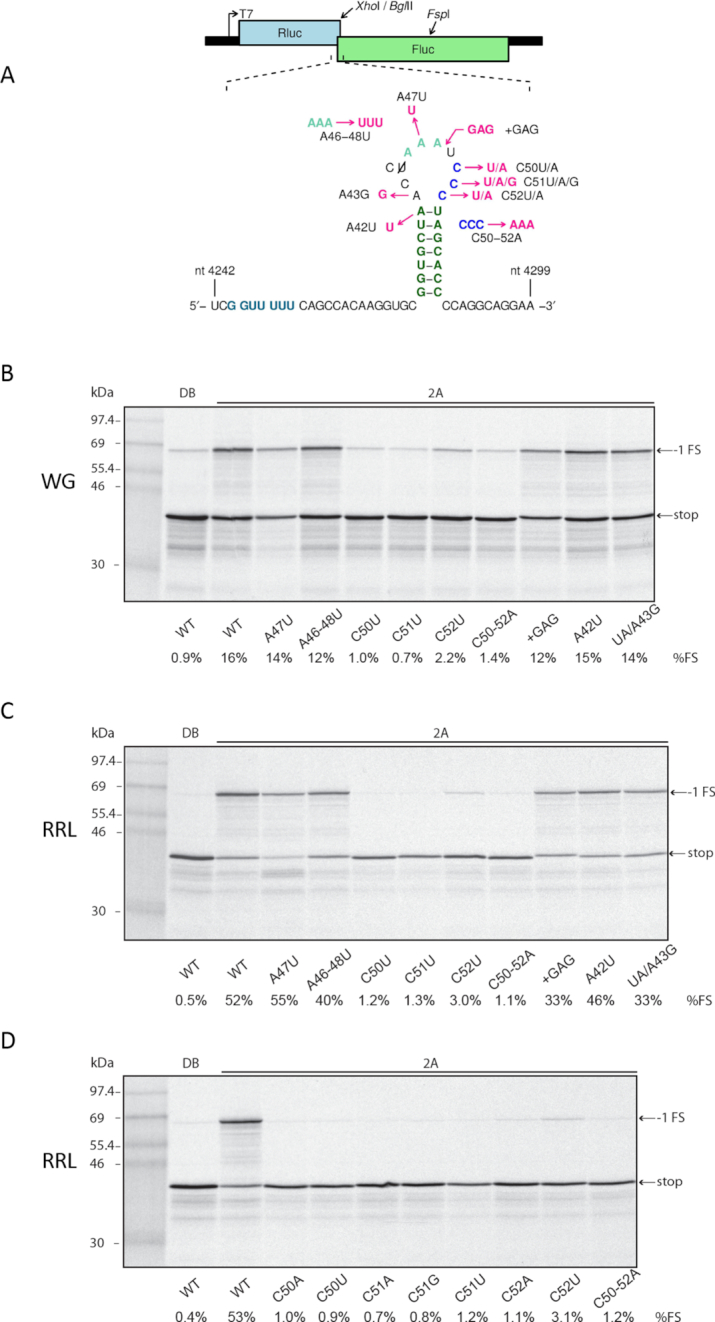
Mutational analysis of the loop region. (**A**) Mutations introduced into the TMEV PRF signal in reporter plasmid pDluc. (**B–D**) RNAs containing the TMEV PRF signal were translated in WG (**B**) or RRL (**C, D**) with 1.8 μM recombinant TMEV 2A or with dialysis buffer (DB). Markers are in lane 1.

Previously (before the discovery of the role of 2A in cardiovirus PRF) we analysed a trio of TMEV stem–loop mutants in the context of a dual luciferase reporter construct transfected into BHK-21 cells which were subsequently infected with TMEV (16). In these mutants we altered the 5′ part or the 3′ part of the stem to disrupt base-pairing, or altered both 5′ and 3′ parts together to restore the predicted duplex but with altered base-pairings at the three basal positions. However the TMEV loop region was not analysed, and only a limited analysis has been performed in EMCV (namely mutations to the CCC triplet; (18)). Therefore we performed a mutational analysis of the TMEV loop region (Figure 4A). Mutation of any nucleotide of the CCC triplet (C50U, C51U, C52U, C50–52A) greatly diminished PRF in both WG and RRL (Figure 4B and C). Of these, C52U was consistently less inhibitory than the other mutations. Additional mutations within the CCC triplet (C50A, C51A, C51G, C52A; tested only in RRL) also nearly completely abolished PRF (Figure 4D). In contrast, mutating the 5′ adjacent AAA triplet (A47U, A46–48U) that is conserved in TMEV and related viruses (Saffold, rat theilovirus) and most, but not all, EMCV isolates (Supplementary Figure S3a of (18)), had little (A47U) or only modest (A46–48U; ∼1.3-fold reduction in both WG and RRL) effect on PRF. Similarly, the mutation A42U which separated the apical base-pair of the duplex had little effect on PRF. Increasing the loop length by 3 nt (+GAG) had only a modest effect on PRF (∼1.3- and ∼1.6-fold reduction in WG and RRL, respectively). We also mutated the first A of the loop to G (UA/A43G) to, potentially, extend the stem duplex by 1 bp by base-pairing with the 3′ C of the CCC triplet (the A43G mutation was performed in the context of the apical base-pair A·U to U·A mutation, termed ‘UA’; see below). Similar to the +GAG mutation, this had little effect on PRF in WG and led to only a ∼1.6-fold reduction in RRL.

### PRF is sensitive to spacer length

**Figure 5.**
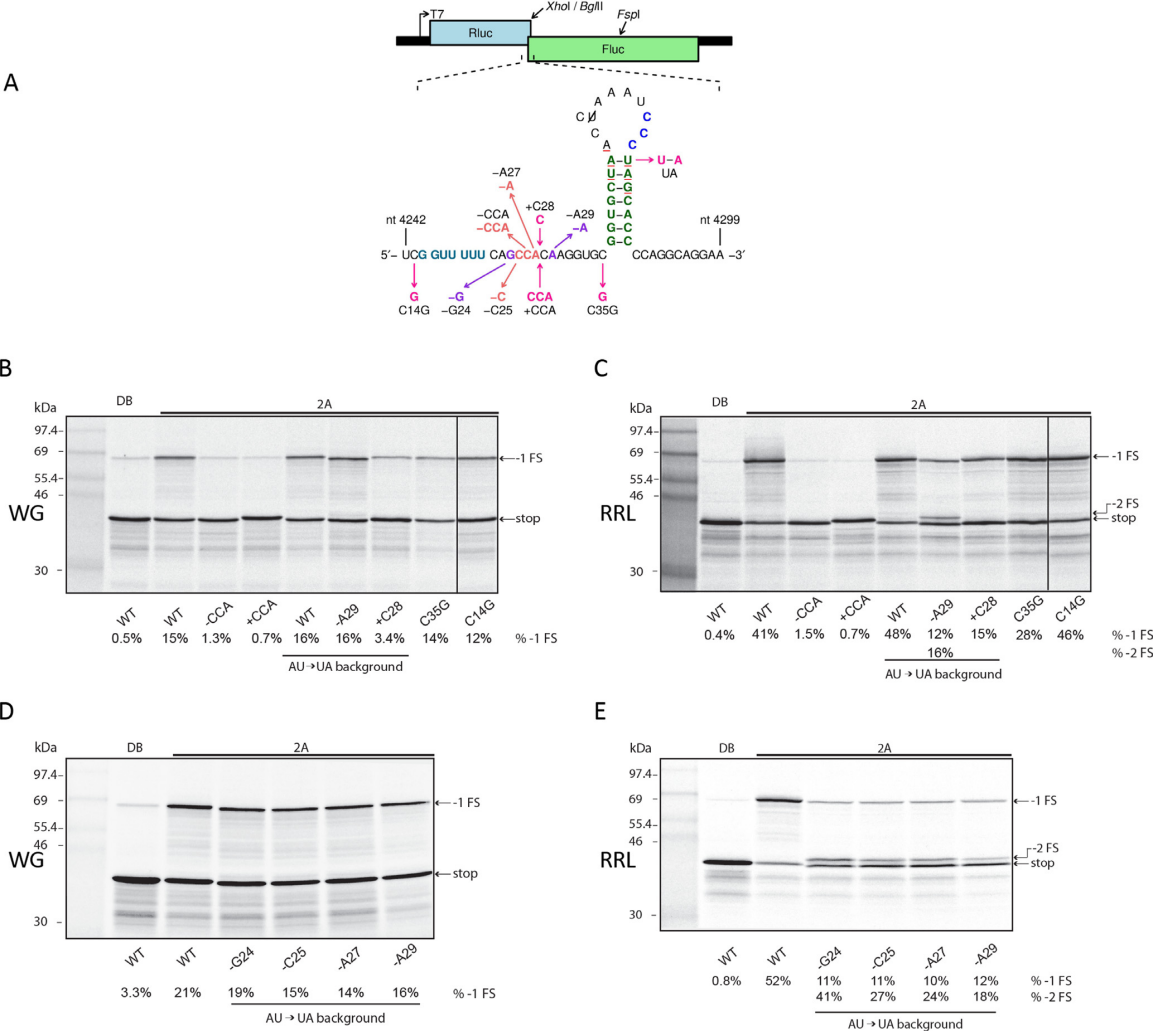
Mutational analysis of the spacer region. (**A**) Mutations introduced into the TMEV PRF signal in reporter plasmid pDluc. (**B–E**) RNAs containing the TMEV PRF signal were translated in WG (**B** and **D**) or RRL (**C** and **E**) with 1.8 μM recombinant TMEV 2A or with dialysis buffer (DB). Markers are in lane 1. The right-most lane in (**B**) and (**C**) was run on the same gel but a single intervening lane has been excised, as indicated by the vertical black line.

The importance of spacer length and nucleotide identity in cardiovirus PRF is poorly studied, restricted so far to the insertion or deletion of a single U nucleotide in the EMCV system (18). To rectify this, we made a number of spacer mutations in the TMEV PRF signal (Figure 5A). Decreasing or increasing the spacer length by 3 nt (−CCA, +CCA) almost abolished PRF (Figure 5B and C). Changing the last nucleotide of the spacer from C to G (C35G), which is predicted to add three extra base-pairs to the base of the stem–loop (5′-UGG-3′ basepairing with 5′-CCA-3′), had little effect on PRF in WG and led to only a ∼1.5-fold reduction in RRL.

Single nucleotide insertions or deletions within the TMEV PRF region are problematic in that they alter the register to bring +1 frame stop codons (UAA and UAG) within the stem–loop into the −1 or 0 frame. To facilitate this analysis, we removed the stop codons by flipping the apical base-pair of the stem from A·U to U·A, a variation which is naturally present in rat theilovirus (e.g. GenBank accession EU542581; see also Supplementary Figure S3A of (18)). This mutation (termed ‘UA’) alone had little effect on PRF (Figure 5B and C; lane 6). Changes in reading frame as a result of spacer mutations were further compensated by additional single-nucleotide insertions or deletions downstream of the stem–loop so that the 0, −1 and −2 frame products would migrate at approximately the same positions for all mutants. In the context of the UA mutation, increasing the spacer length by 1 nucleotide (UA/+C28) reduced −1 PRF 3–5 fold. In RRL, decreasing the spacer by 1 nt (UA/−A29) reduced −1 PRF 4-fold (12%, cf. 48% for UA alone) but also led to a high levels of −2 PRF (16%) (Figure 5C). The appearance of a −2 PRF product is consistent with previous observations of the effect of spacer length mutations in RNA-structure stimulated PRF, where there is potential for (at least) A-site codon:anticodon re-pairing in the −2 frame (31). In contrast, in WG, decreasing the spacer length by a single nucleotide (UA/−A29) had no effect on −1 PRF (16%, cf. 16% for UA alone) and did not lead to noticeable levels of −2 PRF (Figure 5B). Such differences between the *in vitro* translation systems may be a consequence of the slightly smaller footprint of plant ribosomes (32,33). We made additional single-nucleotide deletions, UA/−G24, UA/−C25 and UA/−A27. Similar to the UA/−A29 mutation, these had only a small to modest effect on PRF in WG (Figure 5D) but resulted in a 4–5 fold reduction in −1 PRF and high levels of −2 PRF in RRL (Figure 5E). Interestingly, the UA/−G24 mutant had a substantially higher level of −2 PRF than, for example, the UA/−A29 mutant (41% compared to 18%). It is not clear why this would be the case, but it may be related to effects on chain length from the base composition of the remaining spacer. The UU_UUU section of the TMEV shift site is compatible with A-site re-pairing following a −2 nt shift but the pentanucleotide CG_GUU does not allow obvious P-site re-pairing. We wondered whether the presence of a 5′ pyrimidine (C) might inhibit −2 PRF occurring on the wildtype (WT) PRF signal. Notably, −2 PRF in PRRSV (see Introduction) occurs on an RG_GUU_UUU shift site (R = purine). However when we mutated the 5′ C to G in the TMEV PRF signal (C14G) we did not see appreciable levels of −2 PRF for the WT spacer length (Figure 5B and C).

### TMEV 2A *trans*-activates EMCV PRF but not *vice versa*

**Figure 6.**
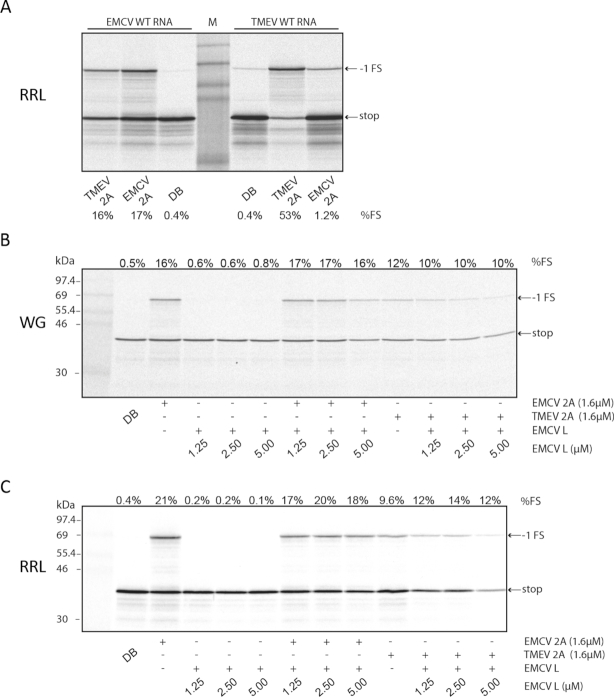
Interchangeability of 2A proteins and lack of activity of L protein in PRF stimulation. (**A**) RNAs containing the EMCV or TMEV PRF signal were translated in RRL with 1.8 μM recombinant EMCV 2A or TMEV 2A or with dialysis buffer (DB). Markers are in the middle lane. (**B**, **C**) RNAs containing the EMCV PRF signal were translated in WG (**B**) or RRL (**C**) with 1.6 μM recombinant EMCV 2A or TMEV 2A, and/or with EMCV L in varying amounts, or with dialysis buffer only (DB). Markers are in the first lane.

The TMEV and EMCV 2A proteins are highly divergent (∼27% aa identity). Moreover, although both stem–loops contain the CCC triplet, the loop regions are otherwise quite different in sequence and in size (21 nt in EMCV, 10 nt in TMEV). Thus we wondered whether the TMEV 2A protein would be able to *trans*-activate PRF on the EMCV PRF signal and/or *vice versa*. RNAs containing the EMCV or TMEV WT PRF signals were translated in RRL in the presence of EMCV or TMEV 2A (Figure 6A). Previously, EMCV 2A was found to stimulate PRF to levels of ∼14–17% at the EMCV PRF signal in RRL and this level was recapitulated here (17%, lane 2). TMEV 2A was able to stimulate similar levels of PRF at the EMCV PRF signal (16%, lane 1). On the other hand, whereas TMEV 2A stimulated efficient PRF at the TMEV PRF signal (53%, lane 6), stimulation by EMCV 2A was only slightly above background (1.2%, lane 7). Thus TMEV 2A can efficiently *trans*-activate PRF at the EMCV PRF signal, but not *vice versa*.

### The viral L protein has no effect on PRF

The viral L protein, encoded at the very N-terminus of the polyprotein is acidic and known to functionally interact with the basic 2A protein during virus infection (34). However, whether L has any effect on PRF has not been investigated. We expressed and purified recombinant EMCV L protein and titrated increasing amounts into WG or RRL cell-free translations programmed with reporter mRNAs containing the WT EMCV PRF signal, with or without added recombinant EMCV or TMEV 2A (Figure 6B and C). As before, EMCV 2A or TMEV 2A on their own stimulated PRF to 10–21%. EMCV L on its own was unable to stimulate PRF. Moreover addition of EMCV L had no appreciable affect on PRF stimulated by TMEV 2A or EMCV 2A in either WG or RRL.

### Mutations in TMEV 2A affect PRF efficiency

Realizing that the interaction between 2A and the RNA stem–loop was likely to involve a basic region on 2A, we previously identified a run of basic residues that is quite well conserved across cardiovirus species (KRIRPFR in the EMCV isolate used in (18), and KGRYRSWKK in the TMEV isolate used herein). Mutating the first two arginines to alanines in EMCV (KRIRPFR to KAIAPFR; R95A/R97A) prevents 2A from binding to the stem–loop (18). However, the effect of 2A mutations on PRF efficiency has not been tested in TMEV.

**Figure 7.**
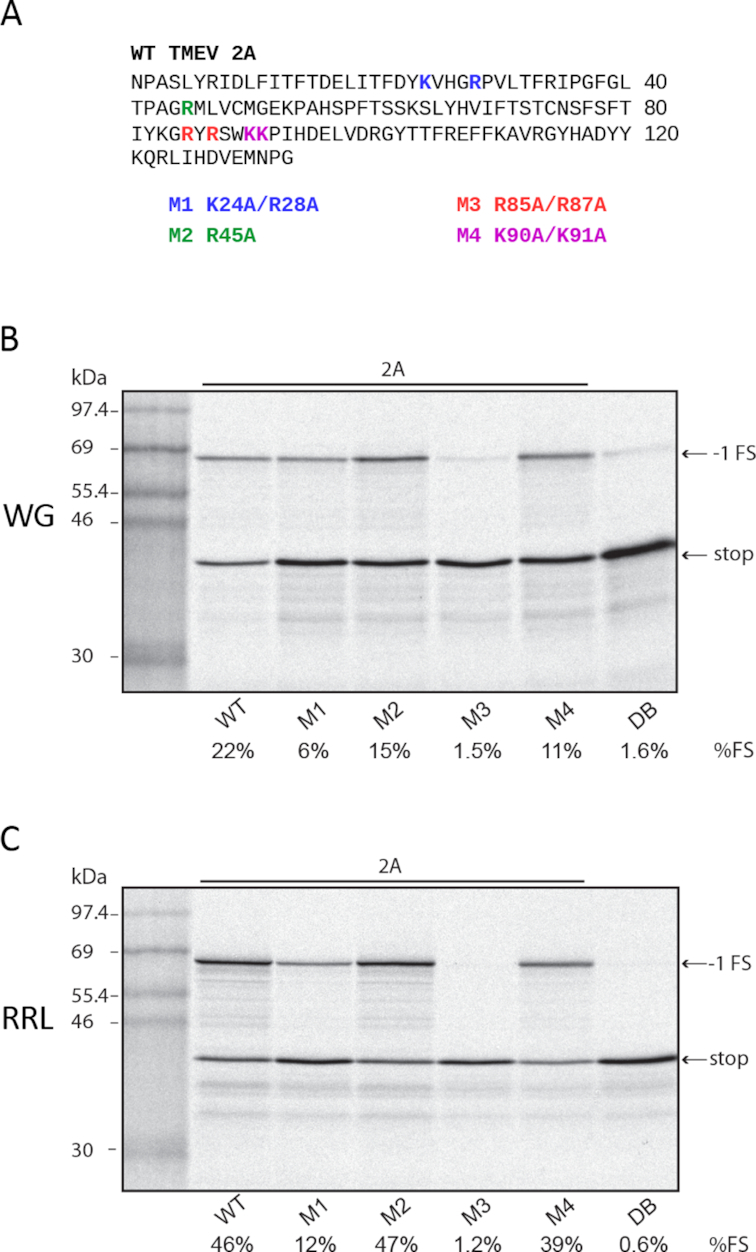
Analysis of TMEV 2A mutants. (**A**) Amino acid sequence of the TMEV 2A protein, with residues that were mutated in M1, M2, M3 and M4 coloured; the corresponding mutations are shown below. (**B, C**) RNAs containing the TMEV PRF signal were translated in WG (**B**) or RRL (**C**) with 1.8 μM recombinant TMEV 2A, 2A mutants M1, M2, M3, M4 or with dialysis buffer (DB). Markers are in the first lane. In this experiment, WT and all mutant 2As were the C-terminally extended versions.

We generated four TMEV 2A mutants targeting different basic residues: M1—K24A/R28A, M2—R45A, M3—R85A/R87A and M4—K90A/K91A (Figure 7A). M3 corresponds to the basic site previously mutated in EMCV 2A. Recombinant mutant 2As were expressed and purified and tested for PRF stimulatory activity (Figure 7B and C). Note that, similar to the EMCV 2A and 2A-mut used in Napthine *et al.* (18), these mutants contained an additional 12 vector-derived C-terminal amino acids (see Methods). In contrast, except where stated otherwise, elsewhere in this study the WT TMEV and EMCV 2As had the authentic C-terminus. To allow a rigorous comparison with the mutants, the C-terminally extended version of WT TMEV 2A was used in Figure 7B and C. For M2 and M4, there was no or only a modest decrease in activity (up to 2-fold). M1 had a 4-fold decrease in activity. Consistent with the previous EMCV data, M3 had greatly reduced activity and was not obviously able to stimulate PRF above background levels. Hereafter, the M3 mutant is referred to as 2A-mut.

### Mutations in 2A or the stem–loop inhibit binding

Mutations in the RNA stem–loop or the 2A protein might inhibit PRF either by reducing or preventing binding between 2A and the stem–loop, or by altering the geometry or other properties of the 2A:stem–loop complex in a way that alters their interaction with the ribosome when the ribosome is positioned on the frameshift site. We used electrophoretic mobility shift assays (EMSAs) to assess the effect of selected mutations on RNA:protein binding. Experiments were performed with recombinant 2A protein and a 59-nt ^32^P-labelled RNA containing the TMEV frameshift signal or a 64-nt ^32^P-labelled RNA containing the EMCV frameshift signal. For the EMSA experiments, the C-terminally extended versions of 2A (see Materials and Methods) were used except where stated otherwise.

**Figure 8.**
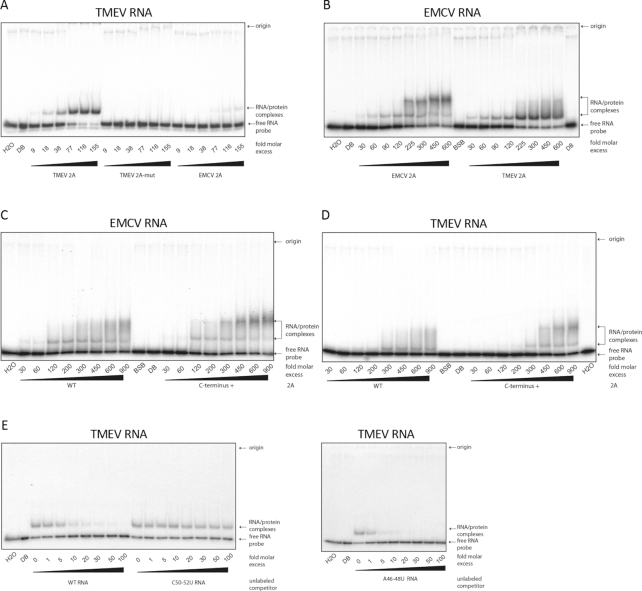
EMSA analysis of cardiovirus 2A RNA binding activity. (**A**) ^32^P-labelled RNA containing the WT TMEV PRF signal was incubated with increasing amounts of TMEV 2A, TMEV 2A-mut, or EMCV 2A and subjected to EMSA on 4% non-denaturing acrylamide gels. (**B**) ^32^P-labelled RNA containing the WT EMCV PRF signal was incubated with increasing amounts of EMCV 2A or TMEV 2A. (**C**) ^32^P-labelled RNA containing the WT EMCV PRF signal was incubated with increasing amounts of EMCV 2A (WT) or the C-terminally extended version of EMCV 2A (C-terminus +). (**D**) ^32^P-labelled RNA containing the WT TMEV PRF signal was incubated with increasing amounts of TMEV 2A (WT) or the C-terminally extended version of TMEV 2A (C-terminus +). (**E**) Unlabelled competitor RNA containing the WT, C50–52U mutant or A46–48U mutant TMEV PRF signal was incubated with ^32^P-labelled RNA containing the WT TMEV PRF signal, and TMEV 2A (0.7 μM), and analyzed by EMSA. In (**A, B, E**), WT and all mutant 2As were the C-terminally extended versions (see Methods). In (**A–D**), numbers below lanes show fold molar excess of 2A with respect to RNA (10 nM). In (**E**), numbers below lanes show fold molar excess of competitor RNA with respect to ^32^P-labelled WT RNA (10 nM). In lanes BSB, DB and H_2_O, RNA was incubated alone with band-shift buffer, protein dilution buffer or water, respectively.

When the WT TMEV RNA was incubated with increasing amounts of WT TMEV 2A an RNA:protein complex could be observed, indicating that TMEV 2A binds the TMEV PRF signal (Figure 8A). In contrast, TMEV 2A-mut was unable to bind the TMEV PRF signal (Figure 8A). EMCV 2A bound the TMEV PRF signal only very weakly (Figure 8A), consistent with its inability to stimulate efficient PRF at the TMEV signal (Figure 6A). In the converse experiment, we found that EMCV 2A efficiently bound the EMCV PRF signal (Figure 8B), consistent with previous results (18). Moreover, TMEV 2A also bound the EMCV PRF signal (Figure 8B), consistent with the ability of TMEV 2A to efficiently stimulate PRF at the EMCV PRF signal (Figure 6A).

To test whether the short C-terminal extension of vector-derived amino acids on the recombinant 2As affected binding to the PRF signal, we compared WT and C-terminally extended EMCV and TMEV 2As in the EMSA analyses (Figure 8C and D). In both cases, the C-terminally extended 2A had no obvious defect in its ability to bind the corresponding PRF signal. Note that, as expected, the RNA:protein complexes for EMSAs performed with the C-terminally extended 2As migrated more slowly than those for EMSAs performed with the 2As that had the authentic C-terminus.

Next we used EMSA competition assays to assess two of the TMEV stem–loop mutants. Non-radioactive WT, C50–52U mutant or A46–48U mutant RNAs were competed against ^32^P-labelled WT RNA for 2A binding. Whereas the WT and A46–48U mutant RNAs were able to efficiently compete with WT RNA—greatly diminishing radiolabelled RNA:protein complexes at increasing molar excess—the C50–52U mutant RNA was unable to compete, indicating that the CCC sequence is important for 2A binding (Figure 8E).

### 2A is required for PRF during TMEV infection

Protein production by WT and shift site mutant (SS) TMEVs has previously been assessed by metabolic labelling (16). This study found that, at late timepoints, WT virus produces much lower quantities of the proteins encoded downstream of the frameshift site than proteins encoded upstream of the frameshift site when compared with the SS mutant virus. Normalization of WT by SS allowed the PRF efficiency in the natural context of virus infection to be estimated as 74–82% by 7 h p.i. Having now identified the stimulatory role of 2A, we repeated this experiment with the addition of 2A-mut, a virus in which we introduced the R85A/R87A mutations into 2A.

**Figure 9.**
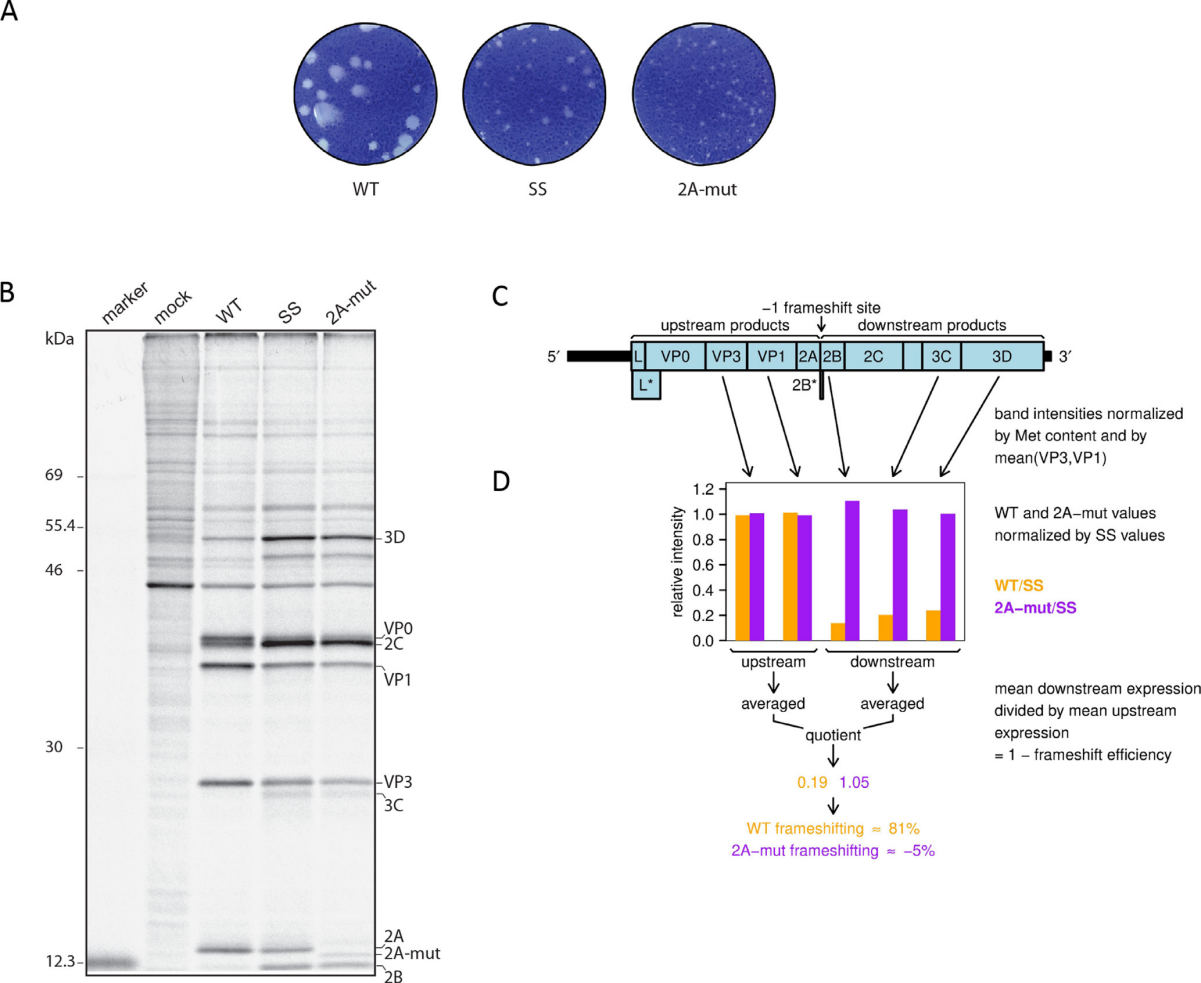
Mutating 2A inhibits frameshifting in TMEV. (**A**) Plaque morphology of WT, SS and 2A-mut viruses on BHK-21 cells. (**B**) Metabolic labelling of BHK-21 cells mock-infected or infected with WT, SS or 2A-mut viruses. Positions of TMEV proteins are indicated. (**C**) Schematic of the TMEV genome. UTRs are indicated in black and CDSs in pale blue. The lengthy 5′ UTR contains an IRES that directs translation of the polyprotein ORF (L-VP0-VP3-VP1-2A-2B-2C-3A-3B-3C-3D), its frameshift truncation (L-VP0-VP3-VP1-2A-2B*), and the overlapping L* ORF. The tiny 2B* protein shares its N-terminal 6 aa with 2B, whereas its C-terminal 8 aa are encoded in the −1 reading frame. (**D**) Ratio of band intensities between WT and SS, or 2A-mut and SS viruses.

We found that the 2A-mut virus is viable but, like the SS mutant virus tested previously, it has a small plaque phenotype (Figure 9A). The protein expression of 2A-mut is similar to SS and both are very different from WT, with much more efficient expression of downstream proteins such as 2B, 2C, 3C and 3D relative to upstream proteins such as VP0, VP3 and VP1 (Figure 9B). The 2A-mut protein migrates a little faster than the WT 2A protein. PRF efficiencies were estimated from the ratio of downstream to upstream expression normalized by the SS mutant (Figure 9C; see Materials and Methods), giving values of 81% for WT virus and −5% for 2A-mut (Figure 9D). (The −5% value illustrates the inherent difficulty in making precise measurements of PRF efficiencies via metabolic labelling.) Thus mutating 2A in the virus context inhibits frameshifting.

### Ribosomes pause at a mutated cardiovirus PRF site

**Figure 10.**
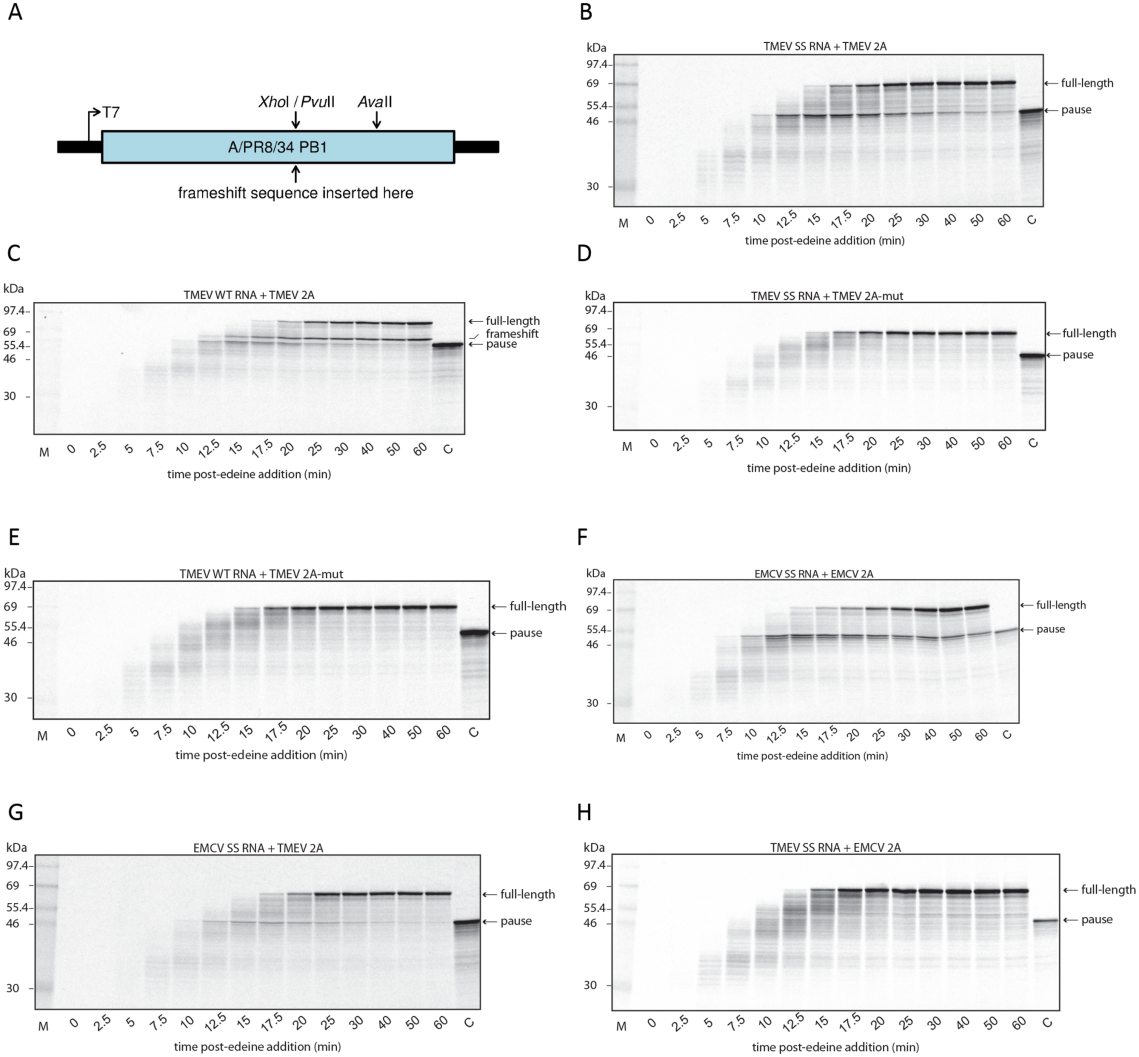
Ribosomal pausing at cardiovirus frameshift signals. (**A**) Wild type (WT) and shift site mutant (SS) TMEV and EMCV frameshift sequences were inserted into reporter plasmid pPS0. (**B**–**H**) RNAs derived from *Ava*II-cut plasmids were translated in WG and, after 5 min, further initiation was halted by the addition of edeine, and aliquots were removed at various times and analyzed by SDS-PAGE. Lanes M and C show markers and the expected size of the ribosomal pause product, respectively. Translations were supplemented with 1.8 μM TMEV 2A, TMEV 2A-mut, or EMCV 2A as indicated. (**B**) TMEV SS RNA + TMEV 2A. (**C**) TMEV WT RNA + TMEV 2A. (**D**) TMEV SS RNA + TMEV 2A-mut. (**E**) TMEV WT RNA + TMEV 2A-mut. (**F**) EMCV SS RNA + EMCV 2A. (**G**) EMCV SS RNA + TMEV 2A. (**H**) TMEV SS RNA + EMCV 2A. As well as the full-length product and the transient pausing product, a frameshift product is produced for TMEV WT RNA in the presence of TMEV 2A only.

Our previous ribosome profiling analysis of EMCV-infected cells showed extensive ribosome pausing (of order 20 s) on a mutated shift site A_GUG_UUU (shift site mutant, SS). In contrast, a much more modest pause was observed on the WT shift site G_GUU_UUU. Greatly enhanced pausing on a mutated shift site was also apparent in an *in vitro* pausing assay using a reporter construct (Figure 10A) containing the EMCV frameshift sequence translated in the presence of recombinant EMCV 2A protein. To test whether these results extend to TMEV, we translated in wheat germ (WG) a reporter construct containing the WT or SS, TMEV or EMCV frameshift sequence in the presence of recombinant TMEV or EMCV 2A (authentic C-terminus) or TMEV 2A-mut (C-terminally extended version). The extent of pausing was assessed by comparing the levels of a translational intermediate corresponding to pausing at the shift site with that of the full-length polypeptide (and also the frameshift product, where applicable). Translation was synchronized by the addition of edeine, a potent inhibitor of initiation, 5 min after the start of the reaction and pausing was monitored over a time course.

Consistent with previous observations for EMCV, we observed a protracted pause at the PRF site when TMEV SS RNA was translated in the presence of TMEV 2A (Figure 10B), but a more transient pause for TMEV WT RNA (Figure 10C). In contrast, no pausing was observed when either RNA was translated in the presence of TMEV 2A-mut (Figure 10D and E). We also tested for cross-activation between EMCV and TMEV, using the shift site mutant RNAs and the WT 2A proteins. As previously seen, translation of EMCV SS RNA in the presence of EMCV 2A leads to a very protracted pause (Figure 10F). TMEV 2A was also able to induce pausing on EMCV SS RNA (Figure 10G); however the pausing induced by TMEV 2A on EMCV SS RNA was much less pronounced than that induced by EMCV 2A. On the other hand, EMCV 2A was unable to induce pausing on TMEV SS RNA (Figure 10H). Thus the pausing assays are consistent with the frameshifting assays, where TMEV 2A can stimulate efficient PRF on EMCV WT RNA but not *vice versa* (Figure 6A).

## DISCUSSION

We have shown that, like EMCV, −1 PRF in TMEV is *trans*-activated by the viral 2A protein. Previous work showed that the efficiency of −1 PRF in EMCV increases from negligible levels at 2 and 4 h p.i. to ∼70% at 6 and 8 h p.i, presumably as a result of increasing cytoplasmic levels of 2A (18). Thus −1 PRF in EMCV plays a dual role in (i) allowing late timepoint expression of the 14 kDa 2B* protein, and (ii) down-regulating enzymatic protein expression by ∼3-fold at late timepoints. In contrast, the 2B* protein in TMEV has only 14 aa, and currently there is no evidence that the peptide is functional (16). Thus, −1 PRF in TMEV may play the singular role of down-regulating enzymatic protein expression at late timepoints. Metabolic labelling indicates that the down-regulation in TMEV may be ∼5-fold—even stronger than in EMCV. As discussed previously, this provides the virus with an elegant solution to build up replication capacity at early timepoints, while favouring structural protein synthesis at late timepoints (18).

Cardiovirus 2A is a multifunctional protein which has no homologue among other picornaviruses. The positively charged region, which is essential for binding to the stem–loop, also doubles as a nuclear localization signal (26,27). Early in infection, 2A is targeted to the nucleus and nucleolus (26). 2A also binds the virus L protein and is thought to shuttle it to the nucleus, where L directs hyperphosphorylation of nuclear pore proteins resulting in inhibition of active nucleocytoplasmic trafficking (34–36). At later timepoints, 2A accumulates in the cytoplasm where it exhibits a diffuse distribution (26). 2A also plays a role in shutoff of cap-dependent translation although the mechanism remains elusive (26,27). A portion of 2A also associates with cytoplasmic 40S but not 80S ribosomes (41). More than 40 years ago, 2A was shown to exhibit non-specific RNA binding activity (37), and we now show that it has enhanced specificity for the cardiovirus PRF stem–loop with a CCC loop triplet being a key part of the recognition motif.

Our targeted 2A mutation that prevented binding of 2A to the stem–loop resulted in a protein expression pattern in the context of virus infection similar to the SS mutant (Figure 9B). Specifically, at late timepoints the replication proteins are expressed at a much higher level relative to the structural proteins for the mutant viruses in comparison to WT virus. With the benefit of hindsight, similar protein expression patterns can be seen in the literature for EMCV mutants in which large parts of 2A have been deleted (e.g. (38,39)). However, deletions in EMCV 2A may also lead to an impairment of VP0-VP3-VP1-2A processing (38,39), which complicates interpretation of these earlier results. TMEV mutants with deletions in 2A—one involving just 11 aa encompassing the conserved basic region altered in our M3 mutant—have been reported and, although virus protein expression patterns were not shown, both mutant viruses were found to be viable but attenuated in BHK-21 cells and were avirulent in mice (40).

The higher *in vitro* −1 PRF levels observed for the TMEV signal (∼20% in WG, ∼58% in RRL) compared to the EMCV signal (16–21% in WG, 14–20% in RRL; (18)) could be due to a more compact stem–loop less vulnerable to differences that might affect RNA folding *in vitro*, or simply that the TMEV −1 PRF signal naturally induces higher PRF levels. The large difference between WG (∼20%) and RRL (∼58%) for the TMEV PRF signal is likely related to structural differences between plant and animal ribosomes (e.g. plant ribosomes have a slightly smaller footprint than mammalian ribosomes) which may affect the geometry of the interaction between the ribosome and the 2A:stem–loop complex; though in principle it could also be due to other host factors (e.g. host proteins) being involved. In contrast, much smaller differences were seen between WG and RRL for the EMCV system (16–21% compared to 14–20%; (18)). The larger size of the EMCV stem–loop and the EMCV 2A protein may be responsible for this different behaviour. By analogy, in arterivirus protein-stimulated PRF, the efficiency of PRF (both −1 and −2) is sensitive to whether PCBP1 or PCBP2 is used as the stimulator, and relative efficiencies also vary between WG and RRL systems (15).

*In vitro* reconstitution of −1 PRF at the cardiovirus signal requires solely the addition of 2A, indicating that no other viral proteins are essential for PRF stimulation. In particular, the L protein (see above) appears to have no effect on PRF. The ability to reconstitute PRF in WG, despite high divergence between plant and animal proteomes, also suggests that no additional host proteins are involved. In contrast, arterivirus protein-stimulated PRF can only be reconstituted in WG with the addition of both the viral *trans*-activator (nsp1β) and mammalian PCBP (15). Further, in previous RiboTrap assays using the EMCV PRF signal and EMCV infected cell lysates, no other proteins were observed to co-immunoprecipitate with the stem–loop at levels similar to 2A (Supplementary Figure S6A of (18)). The reason for the significantly lower PRF efficiency observed *in vitro* (∼20% in WG; ∼58% in RRL) compared to virus infection (∼81%) remains unknown, but possibilities include incomplete activity of the recombinant 2A, effects of more distal RNA sequences, or differences in translational environment such as salt, temperature, pH and ribosome loading.

These experiments enhance our understanding of the cardiovirus PRF signal—one of only two currently known cases of protein-stimulated PRF. The work provides a platform for future biophysical and structural studies, with the TMEV version of cardiovirus PRF being particularly amenable to further study due to the higher *in vitro* PRF efficiency (56% in RRL) and more compact nature of the stem–loop when compared to EMCV. Currently there are no known cellular examples of protein-stimulated PRF; however the regulatory dimension afforded to PRF by using a protein stimulator means that such mechanisms have the potential to play roles in development and homeostasis. An increased understanding of viral cases may help elucidate the potential for cellular cases.

## Supplementary Material

gkz503_Supplemental_File
