# Supplementary material for: Characterization of the stimulators of protein-directed ribosomal frameshifting in Theiler's murine encephalomyelitis virus
Source: Nucleic Acids Res. 2019 Jun 10;47(15):8207–23. doi: 10.1093/nar/gkz503 (PMC6735917; doi:10.1093/nar/gkz503)
Supplement: gkz503_Supplemental_File [file gkz503_supplemental_file.docx]

**Supplementary Information**

**Characterization of the stimulators of protein-directed ribosomal frameshifting in Theiler's murine encephalomyelitis virus**

Sawsan Napthine, Susanne Bell, Chris H. Hill, Ian Brierley*, Andrew E. Firth*

Division of Virology, Department of Pathology, Addenbrooke’s Hospital, University of Cambridge, Cambridge, UK.

* Correspondence to I.B. (email: ib103@cam.ac.uk) or to A.E.F. (aef24@cam.ac.uk)

**Data analysis**

Ribosomal frameshifting assays were carried out in wheat germ (WG) or rabbit reticulocyte lysates (RRL). Each frameshifting assay was performed at least three times. Images and quantifications for representative gel autoradiographs are shown in the main text. Relative values were highly consistent. As an example, triplicate samples from the experiments of Figure 7 are shown in panel A (gel autoradiographs; DB is dilution buffer) and panel B (% –1 PRF values ± SEM; *p*-values versus WT, two-tailed *t*-test with equal variances).

*
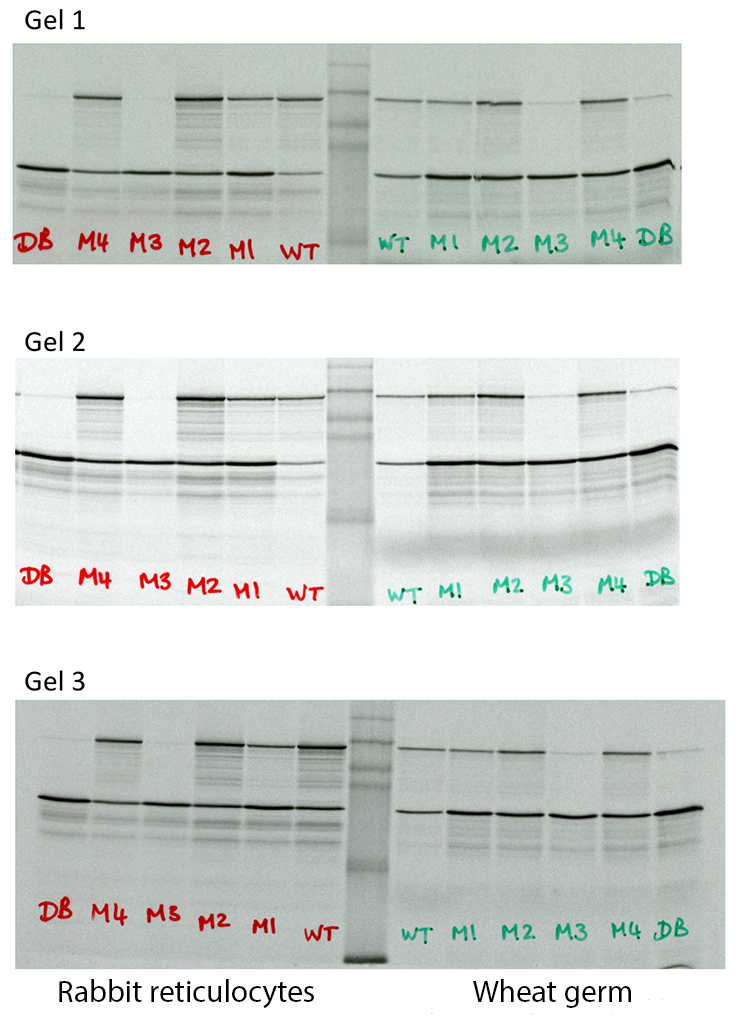
*

*Panel A*

*Panel B*

| **mRNA** | **Details** | **Measured –1 PRF (%)** | | | **Average**  ± **SEM (%)** | ***p*-value (vs WT WG or RRL)** |
| --- | --- | --- | --- | --- | --- | --- |
|  |  | Gel 1 | Gel 2 | Gel 3 |  |  |
| TMEV/WT | WG + 2A | 22.1 | 25.2 | 22.1 | 23.1±1.03 | - |
| TMEV/M1 | WG + 2A | 4.9 | 6.0 | 6.3 | 5.7±0.43 | *p*=0.00010 |
| TMEV/M2 | WG + 2A | 14.8 | 18.4 | 15.4 | 16.2±1.11 | *p*=0.01031 |
| TMEV/M3 | WG + 2A | 1.6 | 1.3 | 1.5 | 1.5±0.09 | *p*=0.00003 |
| TMEV/M4 | WG + 2A | 16.6 | 14.7 | 11.1 | 14.1±1.61 | *p*=0.00932 |
| TMEV/WT | WG + DB | 2.1 | 1.9 | 1.6 | 1.9±0.15 | *p*=0.00003 |
|  |  |  |  |  |  |  |
| TMEV/WT | RRL + 2A | 56.7 | 44.7 | 46.4 | 49.3±3.75 | *-* |
| TMEV/M1 | RRL + 2A | 12.4 | 12.2 | 12.1 | 12.2±0.09 | *p*=0.00059 |
| TMEV/M2 | RRL + 2A | 48.7 | 44.6 | 47.4 | 46.9±1.21 | *p*=0.58036 |
| TMEV/M3 | RRL + 2A | 0.6 | 0.5 | 1.2 | 0.8±0.22 | *p*=0.00021 |
| TMEV/M4 | RRL + 2A | 32.5 | 36.8 | 39.3 | 36.2±1.99 | *p*=0.03693 |
| TMEV/WT | RRL + DB | 0.6 | 0.7 | 0.6 | 0.6±0.03 | *p*=0.00020 |
